# High Azole Resistance in *Aspergillus fumigatus* Isolates from Strawberry Fields, China, 2018

**DOI:** 10.3201/eid2601.190885

**Published:** 2020-01

**Authors:** Yong Chen, Fengshou Dong, Jingya Zhao, Hong Fan, Chunping Qin, Runan Li, Paul E. Verweij, Yongquan Zheng, Li Han

**Affiliations:** Chinese People’s Liberation Army Center for Disease Control and Prevention, Beijing, China (Y. Chen, J. Zhao, H. Fan, C. Qin, L. Han);; Chinese Academy of Agricultural Sciences, Beijing (F. Dong, R. Li, Y. Zheng);; Radboud University Medical Center, Nijmegen, the Netherlands (P.E. Verweij)

**Keywords:** *Aspergillus fumigatus*, azole fungicides, antifungal drug resistance, strawberries, strawberry fields, epidemiology, China, *cyp51A*, azole resistance, agriculture, farms, resistance hotspot, fungi, asexual reproduction, asexual sporulation, MAT1-1, STR type, short tandem repeat typing, antimicrobial resistance

## Abstract

In 2018, we conducted a cross-sectional study to investigate azole resistance in environmental *Aspergillus fumigatus* isolates obtained from different agricultural fields in China. Using 63 soil cores, we cultured for azole-resistant *A. fumigatus* and characterized isolates by their *cyp51A* gene type, short tandem repeat genotype, and mating type. Of 206 *A. fumigatus* isolates, 21 (10.2%) were azole resistant. Nineteen of 21 had mutations in their *cyp51A* gene (5 TR34/L98H, 8 TR34/L98H/S297T/F495I, 6 TR46/Y121F/T289A). Eighteen were cultured from soil samples acquired from strawberry fields, suggesting this soil type is a potential hotspot for azole resistance selection. Twenty resistant isolates were mating type MAT1-1, suggesting asexual sporulation contributed to their evolution. Prochloraz, difenoconazole, and tebuconazole were the most frequently detected fungicides in soil samples with azole-resistant fungus. Our study results suggest that managing the fungicides used in agriculture will help contain the problem of antifungal drug resistance in clinics.

Triazoles are among the main class of drugs used for the treatment of invasive and chronic aspergillosis (*1*,*2*). However, the effectiveness of this drug class is being threatened by the emergence and global spread of azole resistance in clinical and environmental *Aspergillus fumigatus* isolates (*3*,*4*). Resistance is believed to develop predominantly through 2 distinct routes: long-term clinical azole therapy and the environmental application of azole fungicides, some of which have been shown to have molecular targets identical to those of medical triazoles and have activity against *A. fumigatus* (*4*,*5*). The main resistance mechanism of *A. fumigatus* involves point mutations in *cyp51A* (gene encoding the protein targeted by antifungal azoles) with or without a tandem repeat (TR) insertion in its promoter (*6*). Two *cyp51A* variants believed to be associated with environmental resistance selection, TR34/L98H and TR46/Y121F/T289A, are highly prevalent worldwide, although the frequency of these resistance alleles varies considerably from country to country (<5%–30%) (*4*,*7*–*9*). Differences in these reported resistance frequencies could be caused by the study design (i.e., the sampling strategy, number of colonies analyzed, and choice of denominator). On the other hand, the reported variances might instead reflect true differences caused by poorly understood phenomena.

One factor that could be contributing to the variation in resistance allele frequencies is differences in regional azole compound use. The use of azole fungicides provides selective pressure for the development of azole resistance among species in soils. Resistance has been reported in environmental *A. fumigatus* isolates in 2 studies conducted in China, and the prevalence of resistance reported in these studies was 1.4% and 2.1% (*10*,*11*). However, in these studies, the concentration of fungicides in the environment the isolates came from was not measured. Also, whether environmental hotspots exist for resistance selection is unknown. Sites supporting the growth, reproduction, and genetic variation of *A. fumigatus* and containing residual azole fungicides, which can facilitate the emergence, amplification, and spread of triazole resistance mutations, are considered to be potential hotspots for azole resistance (*12*). Here, we describe a cross-sectional study we conducted to investigate azole resistance in *A. fumigatus* isolates in different agricultural fields, identify hotspots of resistance, and evaluate the relationship between azole resistance and use of azole fungicides.

## Methods

### Collection of Soil Samples

During July–August 2018, we collected 63 soil cores from agricultural farms or greenhouses located in 8 cities of China (Harbin, Beijing, Weifang, Nanjing, Wuhan, Hangzhou, Yichun, and Loudi; Appendix Figure). We acquired soil cores (to a depth of 20 cm) near rice, watermelon, strawberry, tea leaf, mandarin orange, and vegetable (eggplant, pepper, water spinach, shallot, cabbage, and tomato) (Appendix Table 1) crops using a soil sampler.

### Isolation and Identification of *A. fumigatus* Isolates

We handled and plated samples according to previously described methods (*13*–*15*) with some modifications. In brief, for each soil core, we suspended 2 g of soil from the top (0 cm) and bottom (20 cm) of the column separately into 8 mL of sterile saline with 1% tween and vortexed. We then plated 100 μL of these suspensions on Sabouraud dextrose agar supplemented with chloramphenicol (50 mg/L; Sigma-Aldrich, https://www.sigmaaldrich.com) and incubated at 42°C. We examined plates for *A. fumigatus* growth at 24 h, 48 h, and 72 h. We randomly selected 5 colonies showing *A. fumigatus* morphology for further identification. If the total number of *Aspergillus*-like colonies on the plate was <5, we subcultured them all. We confirmed colonies were *A. fumigatus* isolates by assessing their capacity to grow at 48°C and by sequencing the β-tubulin gene, as previously described (*16*).

### Detection of Residual Fungicide in Soil Samples

We set aside 10 g of soil from the top (0 cm) and bottom (20 cm) of soil cores for residual fungicide analysis. We detected the 6 main fungicides used in agriculture in China (difenoconazole, tebuconazole, epoxiconazole, prochloraz, imazalil, and tricyclazole) using ultra-high-performance liquid chromatography coupled with tandem mass spectrometry by using an Acquity UPLC BEH Column (2.1 mm × 50 mm, 1.7-μm particle size; Waters, https://www.waters.com) (Appendix Table 2), as previously described (*17*). The mobile phase of the column included chromatographically pure methanol (solution A) and 0.2% formic acid (vol/vol) in Milli-Q water (http://www.emdmillipore.com) (solution B), and the flow rate was 0.3 mL/min. We used the following gradient program to detect fungicides with the column: 10% solution A (0 min), 90% solution A (0–1.7 min), 90% solution A (1.7–3.0 min), 10% solution A (3.0–3.1 min), and 10% solution A (3.1–4.0 min).

### Screening of Azole Resistance

Because VIP check screening plates (https://www.vipcheck.nl) are not commercially available in China, we screened *A. fumigatus* isolates for azole resistance using azole-containing 4-well plates that we prepared. In plate wells, we used RPMI 1640 agar medium (Sigma-Aldrich) supplemented with 4 mg/L itraconazole, 2 mg/L voriconazole, 0.5 mg/L posaconazole, or no fungicide (control well), according to European Committee on Antimicrobial Susceptibility Testing (EUCAST) recommendations (*18*). We used 2 azole-resistant isolates (C135 and C02810) from our laboratory (*19*) and 1 azole-susceptible isolate (ATCC 204305; American Tissue Culture Collection, https://www.atcc.org) for quality control purposes. We performed experimental procedures and interpreted results as recommended by EUCAST (*18*).

### Antifungal Drug Susceptibility Testing and *cyp51A* Gene Sequencing

We conducted antifungal drug susceptibility testing for all isolates demonstrating any growth on >1 azole-containing agar plate. We conducted in vitro drug susceptibility testing with 3 clinical azoles (itraconazole, voriconazole, and posaconazole) and 7 azole fungicides used in agriculture (epoxiconazole, bromucanozole, tebuconazole, difenoconazole, propiconazole, imazalil, and prochloraz) using the EUCAST microbroth dilution E. Def 9.3 method (*20*). We used the same drug concentration ranges and methods for quality control as done in our previous study (*19*) and, for confirmed azole-resistant isolates, amplified and sequenced the *cyp51A* gene and its promoter, as described previously (*21*).

### Genotyping of *A. fumigatus* Isolates

For all azole-resistant isolates, we determined cell surface protein (CSP) type and short tandem repeat (STR) type (i.e., type of 9 microsatellite loci [STRAf 2A, 2B, 2C, 3A, 3B, 3C, 4A, 4B, and 4C]) by PCR amplification and sequencing (*22*,*23*). We identified the mating type of all azole-resistant isolates and a randomly selected subset of azole-susceptible isolates using a PCR (with 2 different primer sets) designed to amplify mating type–specific genes (*24*). We genetically characterized the azole-resistant *A. fumigatus* (ARAF) isolates obtained in this study (n = 21) and other studies conducted in China (n = 30) by performing a categorical analysis of the previously mentioned 9 microsatellite markers using the UPGMA clustering in BioNumerics 7.5 (http://www.applied-maths.com). We also analyzed the STR typing data of all ARAF isolates from this study and 580 representative azole-resistant and azole-susceptible isolates from different countries (*19*) and presented the information as a minimum spanning tree of categorical data with default settings.

### Statistical Analysis

We analyzed data with SPSS 19.0 (IBM Corporation, https://www.ibm.com) and used the χ^2^ test to evaluate differences in the prevalence of ARAF isolates by sample type. We considered p values <0.05 statistically significant.

## Results

### Detection of Azole-Resistant *A. fumigatus* Isolates in Soil Samples

From 126 soil sample suspensions cultured for 72 h, we obtained 210 suspected *A. fumigatus* isolates. After further phenotypic and genotypic identification, 206 isolates (140 from topsoil [0 cm] and 66 from deep soil [20 cm]) were identified as *A. fumigatus* sensu stricto (Table 1). After screening for azole resistance on self-prepared 4-well plates, 23 isolates showed the ability to grow on >1 azole-containing agar. Further confirmatory MIC testing showed that 21 *A. fumigatus* isolates were azole resistant according to EUCAST criteria. The total prevalence of azole resistance among all *A. fumigatus* isolates was 10.2% (21/206).

**Table 1:** Prevalence of ARAF isolates in soil samples from different crops, China, 2018*

Crop	Soil depth, cm	No. ARAF-positive soil samples/no. samples (%)	No. ARAF isolates/no. isolates (%)
Watermelon	0	0/10	0/33
	20	0/10	0/13
Rice	0	1/16 (6.3)	1/20 (5.0)
	20	0/16	0/11
Vegetable	0	1/11 (9.1)	2/33 (6.1)
	20	0/11	0/18
Strawberry	0	6/10 (60.0)	16/44 (36.4)
	20	2/10 (20.0)	2/23 (8.7)
Tea leaf	0	0/5	0/6
	20	0/5	0/0
Citrus	0	0/11	0/4
	20	0/11	0/1
Total	0	8/63 (12.7)	19/140 (13.6)
	20	2/63 (3.2)	2/66 (3.0)

*ARAF, azole-resistant *Aspergillus fumigatus*.

Overall, 19 ARAF isolates were obtained from 8 topsoil samples acquired near strawberry, vegetable, and rice plants, and 2 ARAF isolates were obtained from 2 deep soil samples acquired near strawberry plants. The prevalence of ARAF isolates was higher in topsoil samples (13.6% [19/140]) than deep soil samples (3.0% [2/66], χ^2^ = 5.44; p = 0.020). Of 10 soil cores acquired near strawberry plants, ARAF isolates were detected in 6 (60.0%) topsoil samples and 2 (20.0%) deep soil samples. The 8 soil cores positive for ARAF isolates originated from 8 different farms in Nanjing and Hangzhou in eastern China.

### Characterization of Azole-Resistant *A. fumigatus* Isolates

Of 21 ARAF isolates, 17 were resistant to itraconazole (MIC >4 mg/L), 15 were resistant to voriconazole (MIC >4 mg/L), and all were resistant to posaconazole (MIC >0.5 mg/L) (Appendix Table 3). Sequencing of the *cyp51A* gene and its promoter showed that 19 ARAF isolates harbored 3 commonly identified nucleotide and amino acid change combinations, TR34/L98H (n = 5), TR34/L98H/S297T/F495I (n = 8), and TR46/Y121F/T289A (n = 6); no mutations were identified in the remaining 2 ARAF isolates (E2012-0-2 and E2012-0-4). CSP typing showed that all 5 TR34/L98H ARAF variants corresponded to CSP type t02, 7 of 8 TR34/L98H/S297T/F495I ARAF variants corresponded to CSP type t01 or t11, and all 6 TR46/Y121F/T289A ARAF variants corresponded to CSP type t01 or t04A. The results of mating type identification showed that 20 ARAF isolates were MAT1–1, and only 1 isolate (E2006-0-5) was MAT1–2. Among 21 randomly selected azole-susceptible *A. fumigatus* isolates, 12 were MAT1–1 and 9 MAT1–2.

### In Vitro Susceptibility to Azole Fungicides

High MICs of 5 azole fungicides (epoxiconazole, bromucanozol, tebuconazole, difenoconazole, and propiconazole) were required to inhibit the growth of the 19 ARAF isolates with *cyp51A* mutations (Appendix Table 3). For the 2 ARAF isolates that harbored no *cyp51A* mutations (E2012-0-2 and E2012-0-4), reference strain ATCC 204305, and the azole-susceptible *A. fumigatus* isolates in our previous study (*19*), the MICs of all 7 azole fungicides tested were similar. The MICs of the 2 imidazoles (imazalil and prochloraz) were greater for the TR34/L98H/S297T/F495I and TR46/Y121F/T289A ARAF isolates than they were for the TR34/L98H ARAF isolates and the ARAF isolates without *cyp51A* mutations. For the TR34/L98H/S297T/F495I and TR46/Y121F/T289A ARAF isolates, the MICs of prochloraz were >32 mg/L.

### Detection of Residual Azole Fungicide in Soil Samples

Of the 6 azole fungicides used in agriculture that we tested for, difenoconazole, prochloraz, and tebuconazole were the most frequently detected; epoxiconazole and imazalil were not detected in any soil samples (Table 2). Of the 10 topsoil samples acquired from strawberry-planted fields, difenoconazole (0.0104–0.0385 mg/kg) was detected in 8 and prochloraz (0.0116–0.05 mg/kg) in 7 (Appendix Table 1). We also detected prochloraz in 3 soil samples from 2 sampling sites of vegetable-planted fields. Tebuconazole was detected in 23 of the 32 topsoil and deep soil samples acquired from rice-planted fields. Prochloraz (0.0115–0.05 mg/kg) was detected in 6 of 8 ARAF-positive topsoil samples, difenoconazole (0.0115–0.0385 mg/kg) in 5 of 8, and tebuconazole (0.015–0.0805 mg/kg) in 3 of 8. No azole fungicides were detected in the 2 ARAF-positive deep soil samples. Many azole fungicides, including prochloraz and difenoconazole, had been actively used by the farmers of the fields that we sampled to control for disease during seasons of high temperatures.

**Table 2:** Fungicides detected in soil samples acquired near different crops, China, 2018

Crop	Soil depth, cm	No. soil samples	No. (%) samples containing fungicide*					
			Difenoconazole	Prochloraz	Tebuconazole	Epoxiconazole	Imazalil	Tricyclazole
Watermelon	0	10	0	0	1 (10.0)	0	0	0
	20	10	0	0	0	0	0	0
Rice	0	16	0	1 (6.3)	12 (75.0)	0	0	4 (25.0)
	20	16	0	1 (6.3)	11 (68.8)	0	0	0
Vegetable	0	11	1 (9.1)	3 (27.3)	1 (9.1)	0	0	0
	20	11	0	0	0	0	0	0
Strawberry	0	10	8 (80.0)	7 (70.0)	3 (30.0)	0	0	0
	20	10	1 (10.0)	1 (10.0)	1 (10.0)	0	0	0
Tea leaf	0	5	0	0	0	0	0	0
	20	5	0	0	0	0	0	0
Citrus	0	11	0	0	0	0	0	0
	20	11	0	0	0	0	0	0
Total	0	63	9 (14.3)	11 (17.5)	17 (27.0)	0	0	4 (6.3)
	20	63	1 (1.6)	2 (3.2)	12 (19.0)	0	0	0

*Detection limit for all 6 fungicides was 0.01 mg/kg.

### Genetic Characterization of Azole-Resistant *A. fumigatus* Isolates

**Figure 1:**
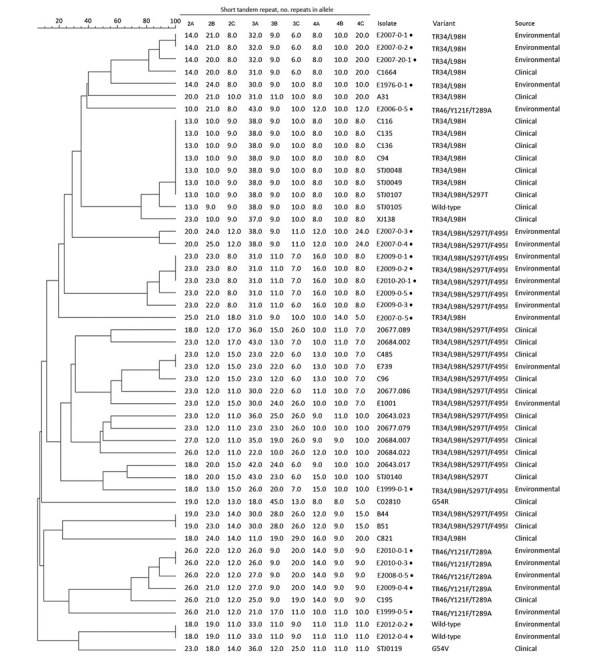
Genotypes of 21 azole-resistant *Aspergillus fumigatus* isolates obtained from farm soils in China, 2018 (black dots), and other azole-resistant *A. fumigatus* isolates from China. This dendrogram was constructed on the basis of a categorical analysis of 9 microsatellite markers (short tandem repeats 2A–4C) by using the UPGMA. Scale bar indicates percentage identity.

ARAF isolates with *cyp51A* mutations had a high diversity of STR types. We observed a close genetic relationship for 5 TR34/L98H/S297T/F495I isolates obtained from strawberry fields of 2 different farms in Hangzhou (Figure 1). The 4 TR46/Y121F/T289A variants isolated from strawberry fields of 3 different farms in Hangzhou were also closely related. The 2 ARAF isolates without *cyp51A* mutations (E2012-0-2 and E2012-0-4) were not genetically related to any other isolate from China, except for a clinical isolate with a G54V amino acid change.

**Figure 2:**
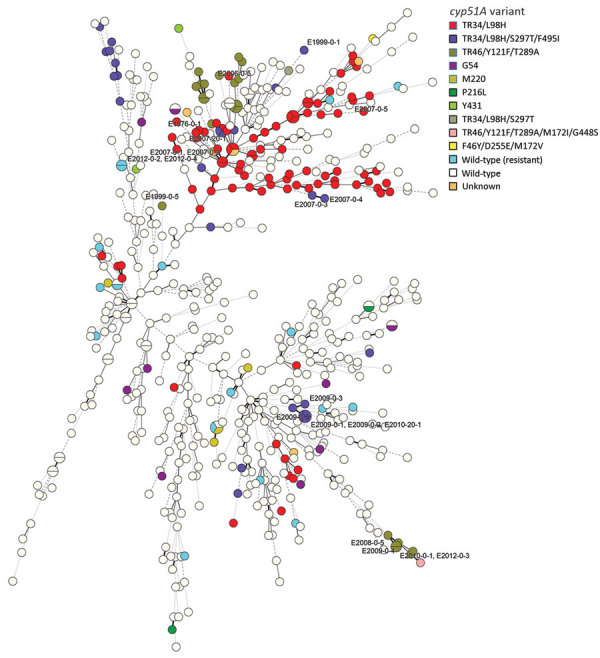
Minimum spanning tree of 21 environmental azole-resistant *Aspergillus fumigatus* (ARAF) isolates, China, 2018 (labeled), and 580 other ARAF and azole-susceptible *A. fumigatus* isolates. The tree was constructed on the basis of short tandem repeat type for all 9 microsatellite markers. Each circle represents 1 unique short tandem repeat genotype but might include multiple *cyp51A* variants. All ARAF isolates from Nanjing are located in the upper right clade of the tree, which represents a major clone complex of ARAF strains disseminated worldwide. All 5 TR34/L98H/S297T/F495I and 4 TR46/Y121F/T289A isolates from Hangzhou are located in the lower right clade of the tree, consisting mainly of azole-susceptible *A. fumigatus* isolates.

We evaluated the population structure of 601 worldwide *A. fumigatus* isolates on the basis of their STR type (Figure 2). All of the ARAF isolates from Nanjing were part of the major clone complex of ARAF strains disseminated widely throughout the world. All 5 TR34/L98H/S297T/F495I isolates and 4 TR46/Y121F/T289A isolates from Hangzhou clustered within a group mainly consisting of azole-susceptible *A. fumigatus* isolates. These findings suggest that the ARAF isolates from Hangzhou and Nanjing originated from different sources.

## Discussion

The rapid dissemination of azole resistance among *A. fumigatus* strains around the world has become an increasing public health problem. An investigation in the Netherlands indicated that an azole resistance mutation (a triple 46-bp repeat in the *cyp51A* promoter) continues to spread in the environment, and compost containing residual azole fungicide was identified as the possible hotspot for this *A. fumigatus* variant (*25*). As of November 2019, a limited number of studies were available on azole resistance among environmental *A. fumigatus* isolates obtained from agricultural fields in China. In 1 study, the prevalence of azole resistance among 73 *A. fumigatus* isolates collected from soils near crops producing vegetables and fruits (such as strawberries, grapes, carrots, watermelons, pumpkins, shallots, luffas, and eggplants) was investigated in greenhouses in Zhejiang Province (*11*). In that study, the authors were able to identify 3 (4.1%) resistant isolates: 1 TR34/L98H/S297T/F495I isolate and 1 TR46/Y121F/T289A isolate from soils near strawberry plants and 1 TR46/Y121F/T289A isolate from soil near a luffa plant. However, the azole fungicide levels in the samples were not investigated, and no TR34/L98H isolate was cultured.

Our study suggests that, in China, ARAF with different *cyp51A* mutations is abundant in strawberry field soils and might be a potential hotspot for the emergence of *A. fumigatus* azole resistance. In a study conducted in the United Kingdom, azole-resistant *A. fumigatus* isolates were identified in several products, including tea and peppers, some of which originated from China (*26*). In this study, we found 2 ARAF isolates in soil sampled near pepper plants. The findings of these 2 studies suggest a high possibility for the transmission of ARAF isolates through international trade, which could pose a great challenge for containing the problem of azole resistance.

We characterized azole resistance of *A. fumigatus* isolates collected at 2 different soil depths, at the surface and 20 cm below the surface. Our results showed that the prevalence of ARAF isolates was much higher in topsoil samples than deep soil samples, a finding potentially attributable to different selective pressures at different soil depths. Compared with the azole fungicide detection rates in 20-cm deep soil samples, the detection rates, particularly for 3 fungicides (difenoconazole, tebuconazole, and prochloraz), in topsoil samples were substantially higher. In the 8 topsoil samples harboring the 19 ARAF isolates, we detected >1 azole fungicide, prochloraz being the most prevalent.

In vitro susceptibility testing showed that the MIC of prochloraz was much higher for *A. fumigatus* TR34/L98H/S297T/F495I isolates than TR34/L98H isolates. This finding is consistent with our previous study (*19*), which suggested that F495I is needed for high imidazole MICs for TR34/L98H/S297T/F495I isolates.

The primary reason for azole fungicide application is not to prevent *A. fumigatus* growth but to prevent green mold, the most destructive postharvest disease of citrus plants caused by *Penicillium digitatum*. Imidazole is the primary fungicide used to control for this disease in China. Surveillance data have shown that imidazole-resistant *P. digitatum* has been isolated from the provinces of Zhejiang, Hubei, and Jiangxi, and the prevalences in these provinces are >30% (*27*,*28*). Alignments of cyp51 protein sequences have shown that F495I in *cyp51A* of *A. fumigatus* corresponds to F506I in *cyp51B* of *P. digitatum*, suggesting that these 2 pathogens harbor similar resistance mechanisms. Therefore, agricultural use of imidazole fungicides might also contribute to the emergence of azole resistance in *A. fumigatus*.

China produces a substantial number of agricultural products and uses a wide array of fungicides for crop protection (*29*). The total amount of fungicides used in agriculture in China was ≈80 million kg/year during 2013–2016, and azole fungicides accounted for more than one third of these fungicides. Triazoles (e.g., tebuconazole) and imidazoles (e.g., prochloraz) are 2 of the most commonly used azole fungicide drug classes. The national registry from the Chinese Ministry of Agriculture showed that, within the azole fungicide class, the usage of tebuconazole and prochloraz almost doubled during 2012–2016. Unlike in countries in Europe, where imidazoles are used less often than triazoles, in China, the frequency of use of imidazoles and triazoles are comparable.

The Chinese Ministry of Agriculture previously released a series of policies on pesticide use (the Zero Growth of Pesticide Usage program) to reduce overuse and inappropriate use of pesticides in agriculture, and the goal of this program was achieved in 2016. Reducing the amount of fungicide used on some crops is likely to happen in China in the near future, which will provide us the opportunity to evaluate the effect of agricultural fungicide use on clinical resistance.

The genetic analysis of ARAF isolates from this study and previous studies provided us information about the emergence of azole resistance in *A. fumigatus* in China. First, nearly all ARAF isolates were MAT1–1, except 1 isolate, E2006-0-5, which was a TR46/Y121F/T289A variant, suggesting that these ARAF isolates mainly evolved and disseminated through asexual sporulation. A possible role for sexual reproduction in the emergence of azole resistance was reported in the study of isolates from compost samples containing residual azole fungicide (*25*). Compost heaps are warm, dark environments low in oxygen and high in carbon dioxide that promote sexual reproduction and thus genetic recombination; hence, sexual reproduction might also facilitate the emergence of azole resistance. However, mating type has rarely been reported in most studies, so a conclusion on the role of sexual and asexual reproduction in azole resistance cannot be drawn. Second, the ARAF variants that we isolated (which harbored different *cyp51A* mutations) were genetically unrelated to each other, suggesting that these isolates might have evolved from different sources. Third, compared with the major ARAF clone complex of strains disseminated worldwide, the genotypes of the ARAF isolates from Hangzhou were closely related to azole-susceptible *A. fumigatus* isolates (Figure 2); this finding suggests that the isolates from Hangzhou might be new strains evolving under the selective pressure of the azole fungicides used in that environment.

In conclusion, we identified strawberry planting sites as potential hotspots for the development of azole resistance in *A. fumigatus* in China. The 3 most common *cyp51A* variants, namely TR34/L98H, TR34/L98H/S297T/F495I, and TR46/Y121F/T289A, which accounted for nearly 90% of all the ARAF isolates in China, might be regarded as the 3 fitness peaks in the fitness landscape of *A. fumigatus* (*30*). ARAF isolates with different *cyp51A* mutations can coexist in the same soil sample. Both triazole and imidazole fungicides might provide the selective pressure for the development of azole resistance in *A. fumigatus*. The management of fungicide use in agricultural fields, especially those serving as potential resistance hotspots, such as strawberry fields, is needed to curb the emergence of antifungal drug resistance in clinics.

Appendix Additional information on high azole resistance in *Aspergillus fumigatus* isolates from strawberry fields, China, 2018.
