# Supplementary material for: High Azole Resistance in Aspergillus fumigatus Isolates from Strawberry Fields, China, 2018
Source: Emerg Infect Dis. 2020 Jan;26(1):81–9. doi: 10.3201/eid2601.190885 (PMC6924917; doi:10.3201/eid2601.190885)
Supplement: Appendix — Additional information on high azole resistance in Aspergillus fumigatus isolates from strawberry fields, China, 2018. [file 19-0885-Techapp-s1.pdf]

Article DOI: https://doi.org/10.3201/eid2601.190885

High Azole Resistance in *Aspergillus fumigatus* Isolates from Strawberry Field, China, 2018

Appendix

Appendix Table 1. Detailed information of the soil samples and detection of six azole fungicides

No.	Crops	Sample ID†	soil depth	Geographical distribution	Sampling date	ARAF positive	Difenoconazole, mg/kg	Prochloraz, mg/kg	Tebuconazole, mg/kg	Epoxiconazole, mg/kg	Imazalil, mg/kg	Tricyclazole, mg/kg
1	Watermelon	E1956	0	Beijing	2018/7/4	No	<0.01	<0.01	<0.01	<0.01	<0.01	<0.01
2	Watermelon	E1956	20	Beijing	2018/7/4	No	<0.01	<0.01	<0.01	<0.01	<0.01	<0.01
3	Watermelon	E1957	0	Beijing	2018/7/4	No	<0.01	<0.01	0.053	<0.01	<0.01	<0.01
4	Watermelon	E1957	20	Beijing	2018/7/4	No	<0.01	<0.01	<0.01	<0.01	<0.01	<0.01
5	Watermelon	E1958	0	Beijing	2018/7/4	No	<0.01	<0.01	<0.01	<0.01	<0.01	<0.01
6	Watermelon	E1958	20	Beijing	2018/7/4	No	<0.01	<0.01	<0.01	<0.01	<0.01	<0.01
7	Watermelon	E1959	0	Beijing	2018/7/4	No	<0.01	<0.01	<0.01	<0.01	<0.01	<0.01
8	Watermelon	E1959	20	Beijing	2018/7/4	No	<0.01	<0.01	<0.01	<0.01	<0.01	<0.01
9	Watermelon	E1960	0	Beijing	2018/7/4	No	<0.01	<0.01	<0.01	<0.01	<0.01	<0.01
10	Watermelon	E1960	20	Beijing	2018/7/4	No	<0.01	<0.01	<0.01	<0.01	<0.01	<0.01
11	Watermelon	E1961	0	Weifang city, Shandong province	2018/7/12	No	<0.01	<0.01	<0.01	<0.01	<0.01	<0.01

No.	Crops	Sample ID†	soil depth	Geographical distribution	Sampling date	ARAF positive	Difenoconazole, mg/kg	Prochloraz, mg/kg	Tebuconazole, mg/kg	Epoxiconazole, mg/kg	Imazalil, mg/kg	Tricyclazole, mg/kg
12	Watermelon	E1961	20	Weifang city, Shandong province	2018/7/12	No	<0.01	<0.01	<0.01	<0.01	<0.01	<0.01
13	Watermelon	E1962	0	Weifang city, Shandong province	2018/7/12	No	<0.01	<0.01	<0.01	<0.01	<0.01	<0.01
14	Watermelon	E1962	20	Weifang city, Shandong province	2018/7/12	No	<0.01	<0.01	<0.01	<0.01	<0.01	<0.01
15	Watermelon	E1963	0	Weifang city, Shandong province	2018/7/12	No	<0.01	<0.01	<0.01	<0.01	<0.01	<0.01
16	Watermelon	E1963	20	Weifang city, Shandong province	2018/7/12	No	<0.01	<0.01	<0.01	<0.01	<0.01	<0.01
17	Watermelon	E1964	0	Weifang city, Shandong province	2018/7/12	No	<0.01	<0.01	<0.01	<0.01	<0.01	<0.01
18	Watermelon	E1964	20	Weifang city, Shandong province	2018/7/12	No	<0.01	<0.01	<0.01	<0.01	<0.01	<0.01
19	Watermelon	E1965	0	Weifang city, Shandong province	2018/7/12	No	<0.01	<0.01	<0.01	<0.01	<0.01	<0.01
20	Watermelon	E1965	20	Weifang city, Shandong province	2018/7/12	No	<0.01	<0.01	<0.01	<0.01	<0.01	<0.01
21	Rice	E1966	0	Harbin city, Helongjiang province	2018/7/15	No	<0.01	<0.01	<0.01	<0.01	<0.01	<0.01
22	Rice	E1966	20	Harbin city, Helongjiang province	2018/7/15	No	<0.01	<0.01	<0.01	<0.01	<0.01	<0.01

No.	Crops	Sample ID†	soil depth	Geographical distribution	Sampling date	ARAF positive	Difenoconazole, mg/kg	Prochloraz, mg/kg	Tebuconazole, mg/kg	Epoxiconazole, mg/kg	Imazalil, mg/kg	Tricyclazole, mg/kg
23	Rice	E1967	0	Harbin city, Helongjiang province	2018/7/15	No	<0.01	<0.01	<0.01	<0.01	<0.01	<0.01
24	Rice	E1967	20	Harbin city, Helongjiang province	2018/7/15	No	<0.01	<0.01	<0.01	<0.01	<0.01	<0.01
25	Rice	E1968	0	Harbin city, Helongjiang province	2018/7/15	No	<0.01	<0.01	<0.01	<0.01	<0.01	<0.01
26	Rice	E1968	20	Harbin city, Helongjiang province	2018/7/15	No	<0.01	<0.01	<0.01	<0.01	<0.01	<0.01
27	Rice	E1969	0	Harbin city, Helongjiang province	2018/7/15	No	<0.01	<0.01	<0.01	<0.01	<0.01	<0.01
28	Rice	E1969	20	Harbin city, Helongjiang province	2018/7/15	No	<0.01	<0.01	<0.01	<0.01	<0.01	<0.01
29	Rice	E1970	0	Harbin city, Helongjiang province	2018/7/15	No	<0.01	<0.01	0.025	<0.01	<0.01	<0.01
30	Rice	E1970	20	Harbin city, Helongjiang province	2018/7/15	No	<0.01	<0.01	<0.01	<0.01	<0.01	<0.01
31	Rice	E1971	0	Chibi city, Hubei province	2018/8/16	No	<0.01	<0.01	0.026	<0.01	<0.01	<0.01
32	Rice	E1971	20	Chibi city, Hubei province	2018/8/16	No	<0.01	<0.01	0.026	<0.01	<0.01	<0.01
33	Rice	E1976	0	Nanjing city, Jiangsu province	2018/7/13	Yes	<0.01	0.0386	0.0805	<0.01	<0.01	<0.01
34	Rice	E1976	20	Nanjing city, Jiangsu province	2018/7/13	No	<0.01	0.011	0.028	<0.01	<0.01	<0.01
35	Rice	E1977	0	Nanjing city, Jiangsu province	2018/7/13	No	<0.01	<0.01	0.063	<0.01	<0.01	0.03
36	Rice	E1977	20	Nanjing city, Jiangsu province	2018/7/13	No	<0.01	<0.01	0.029	<0.01	<0.01	<0.01
37	Rice	E1978	0	Nanjing city, Jiangsu province	2018/7/13	No	<0.01	<0.01	0.027	<0.01	<0.01	<0.01

No.	Crops	Sample ID†	soil depth	Geographical distribution	Sampling date	ARAF positive	Difenoconazole, mg/kg	Prochloraz, mg/kg	Tebuconazole, mg/kg	Epoxiconazole, mg/kg	Imazalil, mg/kg	Tricyclazole, mg/kg
38	Rice	E1978	20	Nanjing city, Jiangsu province	2018/7/13	No	<0.01	<0.01	0.023	<0.01	<0.01	<0.01
39	Rice	E1979	0	Nanjing city, Jiangsu province	2018/7/13	No	<0.01	<0.01	0.028	<0.01	<0.01	<0.01
40	Rice	E1979	20	Nanjing city, Jiangsu province	2018/7/13	No	<0.01	<0.01	0.023	<0.01	<0.01	<0.01
41	Rice	E1980	0	Nanjing city, Jiangsu province	2018/7/13	No	<0.01	<0.01	0.022	<0.01	<0.01	<0.01
42	Rice	E1980	20	Nanjing city, Jiangsu province	2018/7/13	No	<0.01	<0.01	0.022	<0.01	<0.01	<0.01
43	Rice	E1981	0	Nanjing city, Jiangsu province	2018/7/13	No	<0.01	<0.01	0.021	<0.01	<0.01	<0.01
44	Rice	E1981	20	Nanjing city, Jiangsu province	2018/7/13	No	<0.01	<0.01	0.022	<0.01	<0.01	<0.01
45	Rice	E1982	0	Loudi city, Hunan province	2018/8/17	No	<0.01	<0.01	0.023	<0.01	<0.01	0.04
46	Rice	E1982	20	Loudi city, Hunan province	2018/8/17	No	<0.01	<0.01	0.023	<0.01	<0.01	<0.01
47	Rice	E1987	0	Yichuan city, Jiangxi Province	2018/8/18	No	<0.01	<0.01	0.026	<0.01	<0.01	0.044
48	Rice	E1987	20	Yichuan city, Jiangxi Province	2018/8/18	No	<0.01	<0.01	0.026	<0.01	<0.01	<0.01
49	Rice	E1988	0	Yichuan city, Jiangxi Province	2018/8/18	No	<0.01	<0.01	0.031	<0.01	<0.01	<0.01
50	Rice	E1988	20	Yichuan city, Jiangxi Province	2018/8/18	No	<0.01	<0.01	0.026	<0.01	<0.01	<0.01
51	Rice	E1991	0	Yichuan city, Jiangxi Province	2018/8/18	No	<0.01	<0.01	0.023	<0.01	<0.01	0.015
52	Rice	E1991	20	Yichuan city, Jiangxi Province	2018/8/18	No	<0.01	<0.01	0.024	<0.01	<0.01	<0.01
53	Vegetable	E1992	0	Weifang city, Shandong province	2018/7/12	No	<0.01	<0.01	<0.01	<0.01	<0.01	<0.01
54	Vegetable	E1992	20	Weifang city, Shandong province	2018/7/12	No	<0.01	<0.01	<0.01	<0.01	<0.01	<0.01
55	Vegetable	E1993	0	Weifang city, Shandong province	2018/7/12	No	<0.01	<0.01	<0.01	<0.01	<0.01	<0.01
56	Vegetable	E1993	20	Weifang city, Shandong province	2018/7/12	No	<0.01	<0.01	<0.01	<0.01	<0.01	<0.01

No.	Crops	Sample ID†	soil depth	Geographical distribution	Sampling date	ARAF positive	Difenoconazole, mg/kg	Prochloraz, mg/kg	Tebuconazole, mg/kg	Epoxiconazole, mg/kg	Imazalil, mg/kg	Tricyclazole, mg/kg
57	Vegetable	E1994	0	Weifang city, Shandong province	2018/7/12	No	<0.01	<0.01	<0.01	<0.01	<0.01	<0.01
58	Vegetable	E1994	20	Weifang city, Shandong province	2018/7/12	No	<0.01	<0.01	<0.01	<0.01	<0.01	<0.01
59	Vegetable	E1995	0	Weifang city, Shandong province	2018/7/12	No	<0.01	<0.01	<0.01	<0.01	<0.01	<0.01
60	Vegetable	E1995	20	Weifang city, Shandong province	2018/7/12	No	<0.01	<0.01	<0.01	<0.01	<0.01	<0.01
61	Vegetable	E1996	0	Weifang city, Shandong province	2018/7/12	No	0.01	0.026	<0.01	<0.01	<0.01	<0.01
62	Vegetable	E1996	20	Weifang city, Shandong province	2018/7/12	No	<0.01	<0.01	<0.01	<0.01	<0.01	<0.01
63	Vegetable	E1997	0	Weifang city, Shandong province	2018/7/12	No	<0.01	0.054	<0.01	<0.01	<0.01	<0.01
64	Vegetable	E1997	20	Weifang city, Shandong province	2018/7/12	No	<0.01	<0.01	<0.01	<0.01	<0.01	<0.01
65	Vegetable	E1998	0	Nanjing city, Jiangsu province	2018/7/13	No	<0.01	<0.01	0.076	<0.01	<0.01	<0.01
66	Vegetable	E1998	20	Nanjing city, Jiangsu province	2018/7/13	No	<0.01	<0.01	<0.01	<0.01	<0.01	<0.01
67	Vegetable	E1999	0	Nanjing city, Jiangsu province	2018/7/13	Yes	<0.01	0.015	<0.01	<0.01	<0.01	<0.01
68	Vegetable	E1999	20	Nanjing city, Jiangsu province	2018/7/13	No	<0.01	<0.01	<0.01	<0.01	<0.01	<0.01
69	Vegetable	E2000	0	Nanjing city, Jiangsu province	2018/7/13	No	<0.01	<0.01	<0.01	<0.01	<0.01	<0.01
70	Vegetable	E2000	20	Nanjing city, Jiangsu province	2018/7/13	No	<0.01	<0.01	<0.01	<0.01	<0.01	<0.01
71	Vegetable	E2001	0	Nanjing city, Jiangsu province	2018/7/13	No	<0.01	<0.01	<0.01	<0.01	<0.01	<0.01

No.	Crops	Sample ID†	soil depth	Geographical distribution	Sampling date	ARAF positive	Difenoconazole, mg/kg	Prochloraz, mg/kg	Tebuconazole, mg/kg	Epoxiconazole, mg/kg	Imazalil, mg/kg	Tricyclazole, mg/kg
72	Vegetable	E2001	20	Nanjing city, Jiangsu province	2018/7/13	No	<0.01	<0.01	<0.01	<0.01	<0.01	<0.01
73	Vegetable	E2002	0	Nanjing city, Jiangsu province	2018/7/13	No	<0.01	<0.01	<0.01	<0.01	<0.01	<0.01
74	Vegetable	E2002	20	Nanjing city, Jiangsu province	2018/7/13	No	<0.01	<0.01	<0.01	<0.01	<0.01	<0.01
75	Strawberry	E2003	0	Nanjing city, Jiangsu province	2018/7/13	No	<0.01	<0.01	<0.01	<0.01	<0.01	<0.01
76	Strawberry	E2003	20	Nanjing city, Jiangsu province	2018/7/13	No	<0.01	<0.01	<0.01	<0.01	<0.01	<0.01
77	Strawberry	E2004	0	Nanjing city, Jiangsu province	2018/7/13	No	0.0128	0.013	0.0125	<0.01	<0.01	<0.01
78	Strawberry	E2004	20	Nanjing city, Jiangsu province	2018/7/13	No	0.0443	<0.01	<0.01	<0.01	<0.01	<0.01
79	Strawberry	E2005	0	Nanjing city, Jiangsu province	2018/7/13	No	0.0104	0.0116	<0.01	<0.01	<0.01	<0.01
80	Strawberry	E2005	20	Nanjing city, Jiangsu province	2018/7/13	No	<0.01	0.082	0.039	<0.01	<0.01	<0.01
81	Strawberry	E2006	0	Nanjing city, Jiangsu province	2018/7/13	Yes	<0.01	0.042	0.015	<0.01	<0.01	<0.01
82	Strawberry	E2006	20	Nanjing city, Jiangsu province	2018/7/13	No	<0.01	<0.01	<0.01	<0.01	<0.01	<0.01
83	Strawberry	E2007	0	Nanjing city, Jiangsu province	2018/7/13	Yes	0.0115	<0.01	0.0251	<0.01	<0.01	<0.01
84	Strawberry	E2007	20	Nanjing city, Jiangsu province	2018/7/13	Yes	<0.01	<0.01	<0.01	<0.01	<0.01	<0.01
85	Strawberry	E2008	0	Hangzhou city, Zhejiang province	2018/7/15	Yes	0.0188	0.05	<0.01	<0.01	<0.01	<0.01
86	Strawberry	E2008	20	Hangzhou city, Zhejiang province	2018/7/15	No	<0.01	<0.01	<0.01	<0.01	<0.01	<0.01
87	Strawberry	E2009	0	Hangzhou city, Zhejiang province	2018/7/15	Yes	0.0385	0.024	<0.01	<0.01	<0.01	<0.01
88	Strawberry	E2009	20	Hangzhou city, Zhejiang province	2018/7/15	No	<0.01	<0.01	<0.01	<0.01	<0.01	<0.01
89	Strawberry	E2010	0	Hangzhou city, Zhejiang province	2018/7/15	Yes	0.0139	<0.01	<0.01	<0.01	<0.01	<0.01

No.	Crops	Sample ID†	soil depth	Geographical distribution	Sampling date	ARAF positive	Difenoconazole, mg/kg	Prochloraz, mg/kg	Tebuconazole, mg/kg	Epoxiconazole, mg/kg	Imazalil, mg/kg	Tricyclazole, mg/kg
90	Strawberry	E2010	20	Hangzhou city, Zhejiang province	2018/7/15	Yes	<0.01	<0.01	<0.01	<0.01	<0.01	<0.01
91	Strawberry	E2011	0	Hangzhou city, Zhejiang province	2018/7/15	No	0.0114	0.039	<0.01	<0.01	<0.01	<0.01
92	Strawberry	E2011	20	Hangzhou city, Zhejiang province	2018/7/15	No	<0.01	<0.01	<0.01	<0.01	<0.01	<0.01
93	Strawberry	E2012	0	Hangzhou city, Zhejiang province	2018/7/15	Yes	0.0338	0.0216	<0.01	<0.01	<0.01	<0.01
94	Strawberry	E2012	20	Hangzhou city, Zhejiang province	2018/7/15	No	<0.01	<0.01	<0.01	<0.01	<0.01	<0.01
95	Tea leaf	E2013	0	Chibi city, Hubei province	2018/8/16	No	<0.01	<0.01	<0.01	<0.01	<0.01	<0.01
96	Tea leaf	E2013	20	Chibi city, Hubei province	2018/8/16	No	<0.01	<0.01	<0.01	<0.01	<0.01	<0.01
97	Tea leaf	E2014	0	Chibi city, Hubei province	2018/8/16	No	<0.01	<0.01	<0.01	<0.01	<0.01	<0.01
98	Tea leaf	E2014	20	Chibi city, Hubei province	2018/8/16	No	<0.01	<0.01	<0.01	<0.01	<0.01	<0.01
99	Tea leaf	E2015	0	Chibi city, Hubei province	2018/8/16	No	<0.01	<0.01	<0.01	<0.01	<0.01	<0.01
100	Tea leaf	E2015	20	Chibi city, Hubei province	2018/8/16	No	<0.01	<0.01	<0.01	<0.01	<0.01	<0.01
101	Tea leaf	E2016	0	Chibi city, Hubei province	2018/8/16	No	<0.01	<0.01	<0.01	<0.01	<0.01	<0.01
102	Tea leaf	E2016	20	Chibi city, Hubei province	2018/8/16	No	<0.01	<0.01	<0.01	<0.01	<0.01	<0.01
103	Tea leaf	E2017	0	Chibi city, Hubei province	2018/8/16	No	<0.01	<0.01	<0.01	<0.01	<0.01	<0.01
104	Tea leaf	E2017	20	Chibi city, Hubei province	2018/8/16	No	<0.01	<0.01	<0.01	<0.01	<0.01	<0.01
105	Citrus	E2018	0	Loudi city, Hunan province	2018/8/17	No	<0.01	<0.01	<0.01	<0.01	<0.01	<0.01
106	Citrus	E2018	20	Loudi city, Hunan province	2018/8/17	No	<0.01	<0.01	<0.01	<0.01	<0.01	<0.01
107	Citrus	E2019	0	Loudi city, Hunan province	2018/8/17	No	<0.01	<0.01	<0.01	<0.01	<0.01	<0.01

No.	Crops	Sample ID†	soil depth	Geographical distribution	Sampling date	ARAF positive	Difenoconazole, mg/kg	Prochloraz, mg/kg	Tebuconazole, mg/kg	Epoxiconazole, mg/kg	Imazalil, mg/kg	Tricyclazole, mg/kg
108	Citrus	E2019	20	Loudi city, Hunan province	2018/8/17	No	<0.01	<0.01	<0.01	<0.01	<0.01	<0.01
109	Citrus	E2020	0	Loudi city, Hunan province	2018/8/17	No	<0.01	<0.01	<0.01	<0.01	<0.01	<0.01
110	Citrus	E2020	20	Loudi city, Hunan province	2018/8/17	No	<0.01	<0.01	<0.01	<0.01	<0.01	<0.01
111	Citrus	E2021	0	Loudi city, Hunan province	2018/8/17	No	<0.01	<0.01	<0.01	<0.01	<0.01	<0.01
112	Citrus	E2021	20	Loudi city, Hunan province	2018/8/17	No	<0.01	<0.01	<0.01	<0.01	<0.01	<0.01
113	Citrus	E2022	0	Loudi city, Hunan province	2018/8/17	No	<0.01	<0.01	<0.01	<0.01	<0.01	<0.01
114	Citrus	E2022	20	Loudi city, Hunan province	2018/8/17	No	<0.01	<0.01	<0.01	<0.01	<0.01	<0.01
115	Citrus	E2023	0	Yichuan city, Jiangxi Province	2018/8/18	No	<0.01	<0.01	<0.01	<0.01	<0.01	<0.01
116	Citrus	E2023	20	Yichuan city, Jiangxi Province	2018/8/18	No	<0.01	<0.01	<0.01	<0.01	<0.01	<0.01
117	Citrus	E2024	0	Yichuan city, Jiangxi Province	2018/8/18	No	<0.01	<0.01	<0.01	<0.01	<0.01	<0.01
118	Citrus	E2024	20	Yichuan city, Jiangxi Province	2018/8/18	No	<0.01	<0.01	<0.01	<0.01	<0.01	<0.01
119	Citrus	E2025	0	Yichuan city, Jiangxi Province	2018/8/18	No	<0.01	<0.01	<0.01	<0.01	<0.01	<0.01
120	Citrus	E2025	20	Yichuan city, Jiangxi Province	2018/8/18	No	<0.01	<0.01	<0.01	<0.01	<0.01	<0.01
121	Citrus	E2026	0	Yichuan city, Jiangxi Province	2018/8/18	No	<0.01	<0.01	<0.01	<0.01	<0.01	<0.01
122	Citrus	E2026	20	Yichuan city, Jiangxi Province	2018/8/18	No	<0.01	<0.01	<0.01	<0.01	<0.01	<0.01
123	Citrus	E2027	0	Yichuan city, Jiangxi Province	2018/8/18	No	<0.01	<0.01	<0.01	<0.01	<0.01	<0.01
124	Citrus	E2027	20	Yichuan city, Jiangxi Province	2018/8/18	No	<0.01	<0.01	<0.01	<0.01	<0.01	<0.01
125	Citrus	E2028	0	Yichuan city, Jiangxi Province	2018/8/18	No	<0.01	<0.01	<0.01	<0.01	<0.01	<0.01
126	Citrus	E2028	20	Yichuan city, Jiangxi Province	2018/8/18	No	<0.01	<0.01	<0.01	<0.01	<0.01	<0.01

*ARAF, azole-resistant *Aspergillus fumigatus*; ID, identification.

†According to the numbering system in our laboratory, we have numbered the soil samples from 1956 to 2012, it was not indicative of years, as all the samples were collected in 2018.

Appendix Table 2. MRM parameters for detection of selected fungicides

Compounds	Molecular formula	Selected ion	Precursor ion, m/z	Qualifier ion, m/z	Cone voltage, V	Collision energy, V	Dwell time, s
Difenoconazole	$C_{19}H_{17}Cl_2N_3O_3$	$[M+H]^+$	406.10	251.10†	40	38	0.024
				337.10‡	40	25	0.024
Tebuconazole	$C_{16}H_{22}ClN_3O$	$[M+H]^+$	308.20	70.00†	35	33	0.024
				125.10‡	35	38	0.024
Epoxiconazole	$C_{17}H_{13}ClFN_3O$	$[M+H]^+$	330.10	70.00‡	28	35	0.024
				121.00†	28	30	0.024
Prochloraz	$C_{15}H_{16}Cl_3N_3O_2$	$[M+H]^+$	376.10	266.00‡	20	25	0.024
				308.10†	20	17	0.024
Imazalil	$C_{14}H_{14}Cl_2N_2O$	$[M+H]^+$	297.20	159.00‡	35	30	0.024
				69.00b†	35	30	0.024
Tricyclazole	$C_9H_7N_3S$	$[M+H]^+$	190.10	136.00‡	35	35	0.024
				163.00†	35	30	0.024

*MRM, multiple reaction monitoring.

†Represents the quantitative ion transition.

‡Means the qualitative ion transition.

Appendix Table 3. Characterization of 21 azole-resistant *Aspergillus fumigatus* isolates from agricultural soils in China and detection of fungicide residues*

Sample ID	Soil depth, cm	Strain ID†	Type of crop	Geographic location	MIC, mg/L										*cyp51A* mutation	CSP type	Mating type	Detection of 6 fungicide residues in soil samples, mg/kg
					ITC	VRC	POS	BRO	EPO	TEB	PRO	DIF	IMA	PRC				
E1976	0	E1976–0-1	Rice	Nanjing	>16	4	1	16	>32	>32	>32	16	2	1	TR_{34}/L98H	t02	*MAT1–1*	PRC 0.0386, TEB 0.0805
E1999	0	E1999–0-1	pepper	Nanjing	>16	2	1	>32	>32	16	>32	>32	8	>32	TR_{34}/L98H/S297T/F495I	t04A	*MAT1–1*	PRC 0.015
E1999	0	E1999–0-5	pepper	Nanjing	2	>16	1	>32	>32	>32	>32	>32	>32	>32	TR_{46}/Y121F/T289A	t04A	*MAT1–1*	PRC 0.015
E2006	0	E2006–0-5	Strawberry	Nanjing	4	>16	2	>32	>32	>32	>32	>32	>32	>32	TR_{46}/Y121F/T289A	t04A	*MAT1–2*	PRC 0.042, TEB 0.015
E2007	0	E2007–0-1	Strawberry	Nanjing	>16	8	1	>32	>32	>32	>32	>32	4	4	TR_{34}/L98H	t02	*MAT1–1*	DIF 0.0115, TEB 0.0251
E2007	0	E2007–0-2	Strawberry	Nanjing	>16	8	1	>32	>32	>32	>32	>32	4	2	TR_{34}/L98H	t02	*MAT1–1*	DIF 0.0115, TEB 0.0251
E2007	0	E2007–0-3	Strawberry	Nanjing	>16	1	1	>32	>32	32	>32	>32	8	>32	TR_{34}/L98H/S297T/F495I	t01	*MAT1–1*	DIF 0.0115, TEB 0.0251
E2007	0	E2007–0-4	Strawberry	Nanjing	>16	2	2	>32	>32	32	>32	>32	8	>32	TR_{34}/L98H/S297T/F495I	t01	*MAT1–1*	DIF 0.0115, TEB 0.0251
E2007	0	E2007–0-5	Strawberry	Nanjing	>16	8	1	16	>32	32	>32	16	2	1	TR_{34}/L98H	t02	*MAT1–1*	DIF 0.0115, TEB 0.0251
E2007	20	E2007-20-1	Strawberry	Nanjing	>16	8	1	>32	>32	>32	>32	>32	4	2	TR_{34}/L98H	t02	*MAT1–1*	None
E2008	0	E2008–0-5	Strawberry	Hangzhou	2	>16	1	>32	>32	>32	>32	>32	32	>32	TR_{46}/Y121F/T289A	t01	*MAT1–1*	PRC 0.05, DIF 0.0188
E2009	0	E2009–0-1	Strawberry	Hangzhou	>16	8	2	>32	>32	32	>32	>32	16	>32	TR_{34}/L98H/S297T/F495I	t11	*MAT1–1*	PRC 0.024, DIF 0.0385
E2009	0	E2009–0-2	Strawberry	Hangzhou	>16	4	2	>32	>32	32	>32	>32	8	>32	TR_{34}/L98H/S297T/F495I	t11	*MAT1–1*	PRC 0.024, DIF 0.0385

Sample ID	Soil depth, cm	Strain ID†	Type of crop	Geographic location	MIC, mg/L										*cyp51A* mutation	CSP type	Mating type	Detection of 6 fungicide residues in soil samples, mg/kg
					ITC	VRC	POS	BRO	EPO	TEB	PRO	DIF	IMA	PRC				
E2009	0	E2009–0-3	Strawberry	Hangzhou	>16	4	2	>32	>32	32	>32	>32	16	>32	TR_{34}/L98H/S297T/F495I	t11	*MAT1–1*	PRC 0.024, DIF 0.0385
E2009	0	E2009–0-4	Strawberry	Hangzhou	4	>16	0.5	>32	>32	>32	>32	>32	>32	>32	TR_{46}/Y121F/T289A	t01	*MAT1–1*	PRC 0.024, DIF 0.0385
E2009	0	E2009–0-5	Strawberry	Hangzhou	>16	1	1	>32	>32	>32	>32	>32	>32	>32	TR_{34}/L98H/S297T/F495I	t11	*MAT1–1*	PRC 0.024, DIF 0.0385
E2010	0	E2010–0-1	Strawberry	Hangzhou	2	>16	0.5	>32	>32	>32	>32	>32	>32	>32	TR_{46}/Y121F/T289A	t01	*MAT1–1*	DIF 0.0139
E2010	20	E2010-20-1	Strawberry	Hangzhou	>16	1	1	>32	>32	>32	>32	>32	32	>32	TR_{34}/L98H/S297T/F495I	t11	*MAT1–1*	None
E2012	0	E2012–0-2	Strawberry	Hangzhou	>16	1	1	1	2	2	2	1	0.125	0.25	None	t10	*MAT1–1*	PRC 0.0216, DIF 0.0338
E2012	0	E2012–0-3	Strawberry	Hangzhou	8	16	2	>32	>32	>32	>32	>32	>32	>32	TR_{46}/Y121F/T289A	t01	*MAT1–1*	PRC 0.0216, DIF 0.0338
E2012	0	E2012–0-4	Strawberry	Hangzhou	1	>16	0.5	1	2	2	2	0.5	0.125	0.25	None	t01	*MAT1–1*	PRC 0.0216, DIF 0.0338

*European Committee on Antimicrobial Susceptibility Testing MIC breakpoints for resistance of ITC, VRC, and POS were >2mg/L, >2mg/L, and >0.25mg/L, respectively. BRO, bromucanozole; CSP, cell surface protein; DIF, difenoconazole; EPO, epoxiconazole; ID, identification; IMA, imazalil; ITC, itraconazole; POS, posaconazole; PRC, prochloraz; PRO, propiconazole; TEB, tebuconazole; VRC, voriconazole.

†The strain ID is composed of sample ID, soil depth, and serial number.

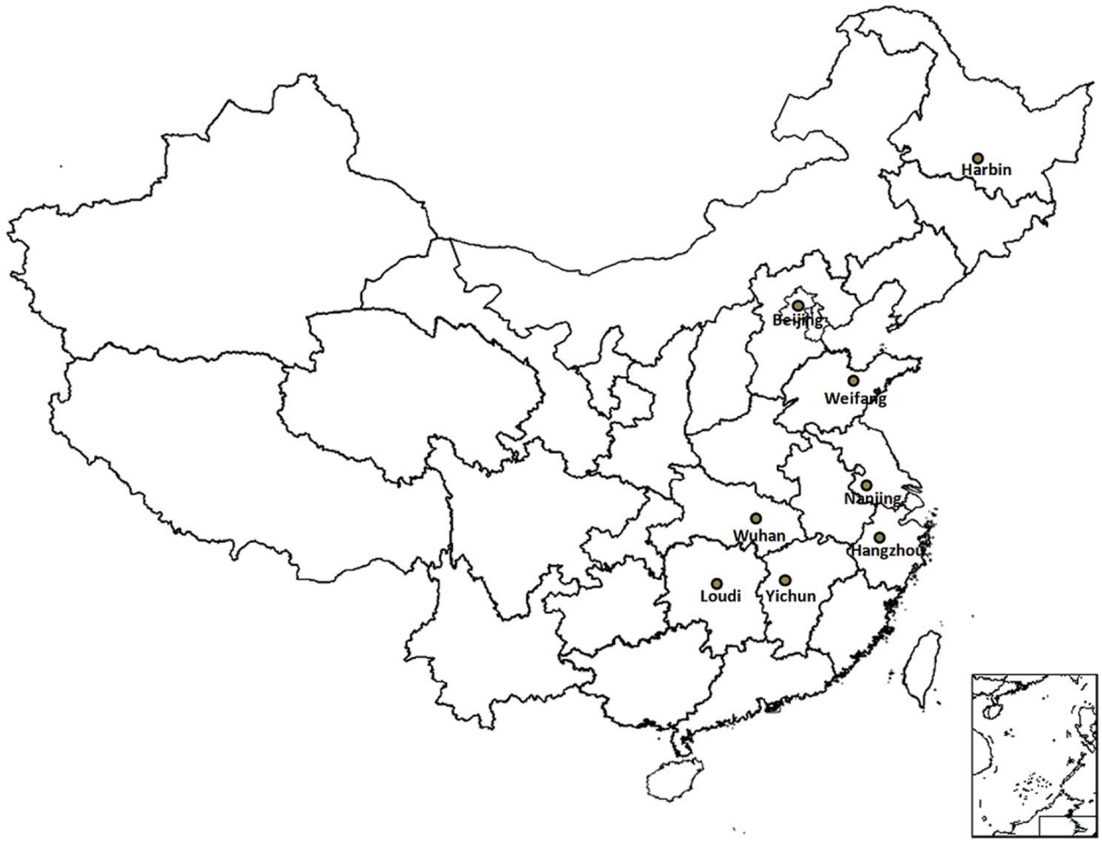

Appendix Figure. Geographic location of soil samples from agricultural farms or greenhouses in eight cities in China.
